# High throughput phenotyping of morpho-anatomical stem properties using X-ray computed tomography in sorghum

**DOI:** 10.1186/s13007-018-0326-3

**Published:** 2018-07-13

**Authors:** Francisco E. Gomez, Geraldo Carvalho, Fuhao Shi, Anastasia H. Muliana, William L. Rooney

**Affiliations:** 1 Department of Crop and Soil Sciences, North Carolina State University, Raleigh, NC 27695 USA; 2 Department of Soil and Crop Sciences, Texas A&M University, 370 Olsen Blvd, College Station, TX 77843 USA; 3 Department of Computer Science and Engineering, Texas A&M University, 3112 TAMU, 710 Ross St, College Station, TX 77843 USA; 4 Department of Mechanical Engineering, Texas A& M University, 401 Joe Routt Blvd, College Station, TX 77843 USA

**Keywords:** X-ray computed tomography, Sorghum, High-throughput phenotyping, Stem morphology, Stem anatomy, Stem biomechanics, Computer vision

## Abstract

**Background:**

In bioenergy/forage sorghum, morpho-anatomical stem properties are major components affecting standability and juice yield. However, phenotyping these traits is low-throughput, and has been restricted by the lack of a high-throughput phenotyping platforms that can collect both morphological and anatomical stem properties. X-ray computed tomography (CT) offers a potential solution, but studies using this technology in plants have evaluated limited numbers of genotypes with limited throughput. Here we suggest that using a medical CT might overcome sample size limitations when higher resolution is not needed. Thus, the aim of this study was to develop a practical high-throughput phenotyping and image data processing pipeline that extracts stem morpho-anatomical traits faster, more efficiently and on a larger number of samples.

**Results:**

A medical CT was used to image morpho-anatomical stem properties in sorghum. The platform and image analysis pipeline revealed extensive phenotypic variation for important morpho-anatomical traits in well-characterized sorghum genotypes at suitable repeatability rates. CT estimates were highly predictive of morphological traits and moderately predictive of anatomical traits. The image analysis pipeline also identified genotypes with superior morpho-anatomical traits that were consistent with ground-truth based classification in previous studies. In addition, stem cross section intensity measured by the CT was highly correlated with stem dry-weight density, and can potentially serve as a high-throughput approach to measure stem density in grass stems.

**Conclusions:**

The use of CT on a diverse set of sorghum genotypes with a defined platform and image analysis pipeline was effective at predicting traits such as stem length, diameter, and pithiness ratio at the internode level. High-throughput phenotyping of stem traits using CT appears to be useful and feasible for use in an applied breeding program.

**Electronic supplementary material:**

The online version of this article (10.1186/s13007-018-0326-3) contains supplementary material, which is available to authorized users.

## Background

Breeding for standability and yield is a major focus of sorghum geneticists and breeders [1, 2]. Stem biomechanical and morpho-anatomical properties affect standability [3–7] and yield components in bioenergy sorghum [8] by influencing the plant’s ability to resist lodging and produce juicy and large stems. However, using existing assays to measure stem biomechanical and morpho-anatomical traits demands significant amounts of labor and time which reduce throughput. New high-throughput and advanced imaging technology provides a solution to alleviate this phenotyping bottleneck [9]. This will ultimately enable plant scientists and breeders to evaluate larger segregating populations which would improve the selection process.

X-ray computed tomography (CT) has become a powerful tool for phenotyping plants and is becoming more widely available to a steadily growing number of plant biologists. As a result, this has led to vast amounts of image data which need to be efficiently managed, processed, mined, and analyzed [10–12]. Despite increasing interest in scanning plant stems using CT [13–15], there have been few studies to visualize and quantify in a high throughput manner above-ground structures of plants using CT.

Plant scientists have been using medical CT and industrial CT scanners to analyze a wide range of extant plant materials [16]. Both scanners are based on the same underlying physics, but due to their difference in applications, industrial CT scanners offer a higher image resolution [17]. Industrial CT scanners are often termed micro-CT or nano-CT because their resolution can range from 5 to 150 µm in the micro-CT and to as low as 0.5 µm in the nano-CT, compared to medical CT scanners, which have at best resolution of 70 µm [18]. However, there are medical scanners available that can obtain similar resolution as industrial CT scanners. Regardless, the type of scanner being utilized, plant scientists are keen for scanners to be as high resolution as possible to accommodate small samples that require a high-resolution scan.

Given their resolution and capacity to detect external and internal phenotypic information in a non-invasive and non-destructive manner, combined with the ability to automate the process, has made the micro-CT the scanner of choice for plant studies [16]. Micro-CT has been successfully used to characterize root structures, developing seeds, stems, leaves, and floral morphology and more at a very detailed level [14, 16–24]. However, depending on the resolution, the size of the sample, and desired signal-to-noise ratio, a CT scan may take several minutes to hours, and there is a sample size tradeoff [11, 17, 21]. Therefore, most studies to date, using micro-CT have been limited to greenhouse studies and used on small samples or small sample sizes that limit the throughput and applicability of this method in a large-scale field-breeding program. Nevertheless, these studies have provided numerous insights and methods to apply CT scanning and image numerous plants tissues.

In clinical research, a combination of biomechanics and X-ray CT has proven to be a powerful research technique to study whole-bone biomechanical properties [25, 26]. Application of such technology in crop improvement could be valuable as well. A study in maize successfully applied an X-ray CT to generate structural morphology of maize stems, which were then implemented in finite-element (FE) analyses. FE analyses performed to study the biomechanical response of these stems discovered that stem strength was highly dependent on stem morphology [27]. The same group using dry maize stems grown under field conditions were able to scan up to 10 samples per run using X-ray CT, and identified a relationship between stem morphology and biomechanics in late-season stem lodging in maize [15].

In sorghum, stem lodging tends to occur at the grain filling stage [28] when there is significant moisture and turgor pressure that may affect biomechanical properties [29]. As tissues mature and subsequently dehydrate as a result of senescence, the modulus of elasticity of these stem increases [3]. Moreover, since bioenergy sorghum stem weight and moisture are good predictors of juice yield [8], it is important to evaluate plants when the physiological influences on the expression of these traits are minimal. Thus, previously mentioned results in late-season stem lodging in maize may not apply to bioenergy sorghum.

The current technological limitations of the micro-CT to acquiring plant morphological and anatomical data makes its application impractical in a field-breeding program. To be useful, the technology must have higher throughput when high-resolution is not needed. To address this problem, we propose the use of a medical CT scanner to visualize and quantify external and internal phenotypic structures in a high-throughput approach that would allow scanning larger samples and increase the number of samples per run of grass stems.

Using a medical CT has several advantages over a micro-CT when high-resolution is not necessary. For example, in a study by du Plessis et al. [30], the authors compared a medical CT to a micro-CT using samples of different densities. The authors concluded the medical CT scanners can produce useful data, significantly reduce scanning time, and provide an alternative for testing large numbers of samples when only moderate resolution is required. Medical CT can also scan larger samples than typical micro-CT systems that would be required to do in parts that would increase scanning time. Thus, using a medical CT would reduce the number of data sets, analysis, and computational power. In addition, industrial micro-CT scanners are not so easily accessible to many crop improvement programs and industrial micro-CT costs run much higher than medical CT systems [30].

Since the ultimate goal of plant biology is to map genotype to phenotype [10], high-throughput genotyping and phenotyping platforms must work in parallel with each other. A robust stem phenotyping platform would mitigate a phenotyping bottleneck existent in bioenergy/forage sorghums. The platform should accurately estimate stem geometry and morpho-anatomical traits, allow for a large number of samples to be run at the same time, fit large samples, produce acceptable repeatabilities for the traits, and work quickly with minimal effort. Thus, the objectives of this study were to (1) develop a practical high-throughput phenotyping platform and image data processing pipeline that can phenotype a large number of samples to extract stem morpho-anatomical properties and (2) validate the methodology.

## Methods

### Plant material

**Fig. 1:**
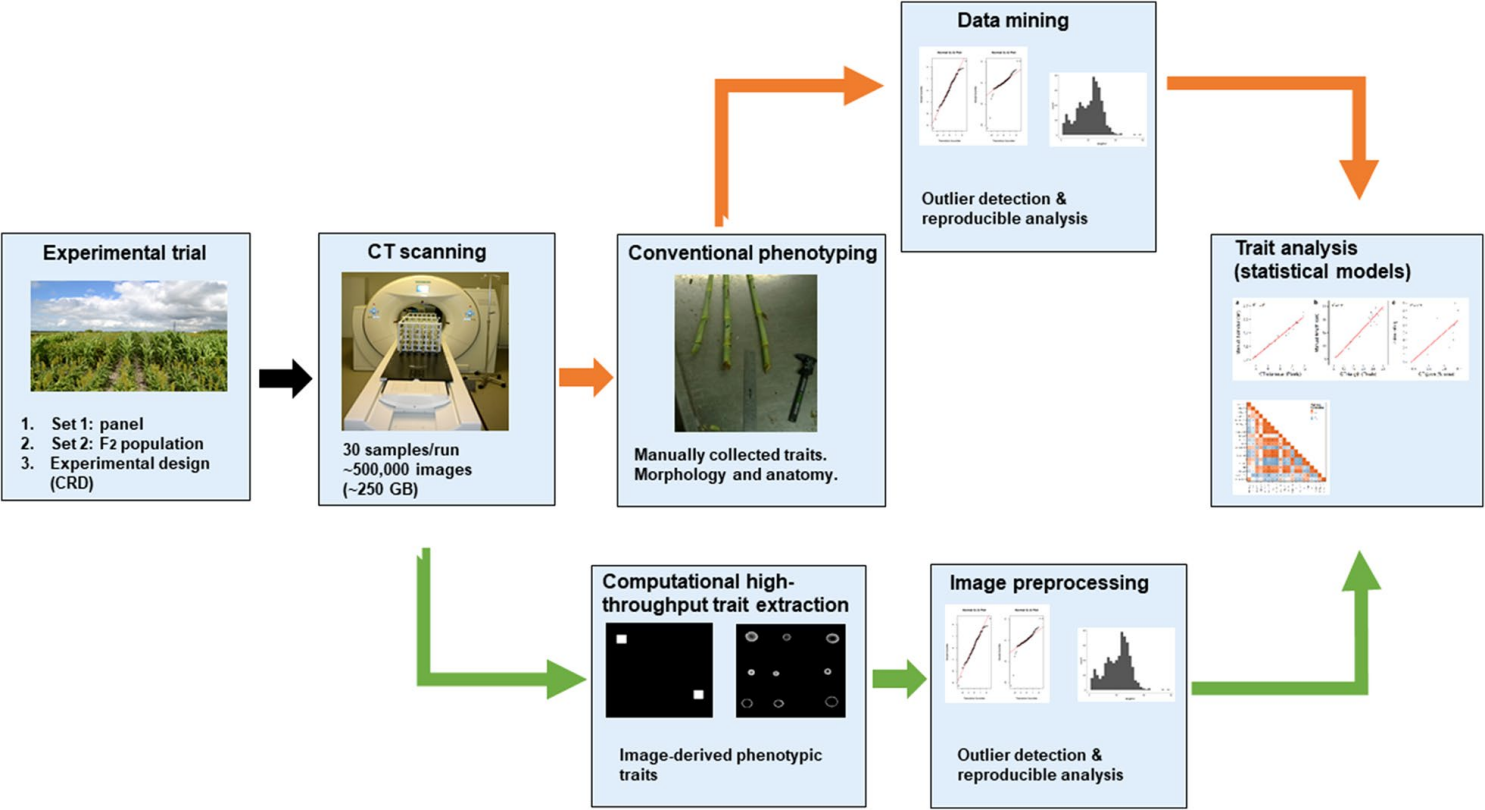
Overview of the high-throughput digital phenotyping imaging pipeline developed in sorghum using X-ray CT (green arrows) and conventional phenotyping (orange arrows) (see “Methods”)

Two different sets of sorghum germplasm were used in the study. Set 1 consisted of 19 genotypes including elite lines and cultivars which contrasted for maturity, stem morphology, stem anatomy, and end-use; while Set 2 consisted of ten F_2_ plants derived from a cross between GIZA114 and Umbrella showing contrasting morpho-anatomical characters (see Additional file 1: Table S1). All genotypes were evaluated to assess the potential of using a medical CT to estimate stem properties in a field-based breeding program. We illustrate the high-throughput digital phenotyping pipeline to quantify morpho-anatomical traits using a medical CT in Fig. 1.

### Experimental details

Two separate field experiments were conducted in 2015 in College Station, Texas (30°33′05.6″N 96°26′14.8″W). Seeds were planted in one-row plots 5 m long and 0.76 m wide. Genotypes from Set 1 were arranged in a complete randomized block design. The target plant density was ~ 75,000 plants ha^−1^. For Set 2, F_2_ seeds were distributed in plots laid out in a row-by-column design. Seeds from Set 1 and Set 2 were sown in April. Agronomic practices standard for sorghum production in this area were used including irrigation as needed to minimize drought stress. Harvesting and evaluations occurred in July, approximately 95 days after planting.

For phenotyping each genotype in Set 1, six healthy plants were randomly selected from the middle of the plot and cut at the soil level. For Set 2, ten F_2_ plants were randomly selected from a ten plot population block. After harvest, any growth taller than 1.5 m was removed to fit the scanner and because stem lodging in sorghum occurs primarily between internodes three and six (which are typically between 0.5 and 1.5 m) [28]. For most samples, the scanned section included internodes 1–7 and some genotypes had > 7 internodes in this section. This procedure was followed by the removal of leaf sheaf across the stem to get precise stem diameter measurements. During this time, samples were kept under moist conditions in a temperature-controlled environment at ~ 20 °C and then transported to be scanned. The process took under an hour after harvesting. After scanning the samples were stored again under the same conditions prior to measurements (maximum 6 h) to prevent tissue dehydration and to maintain natural material properties.

### Phenotyping platform setup and CT-measurement

**Fig. 2:**
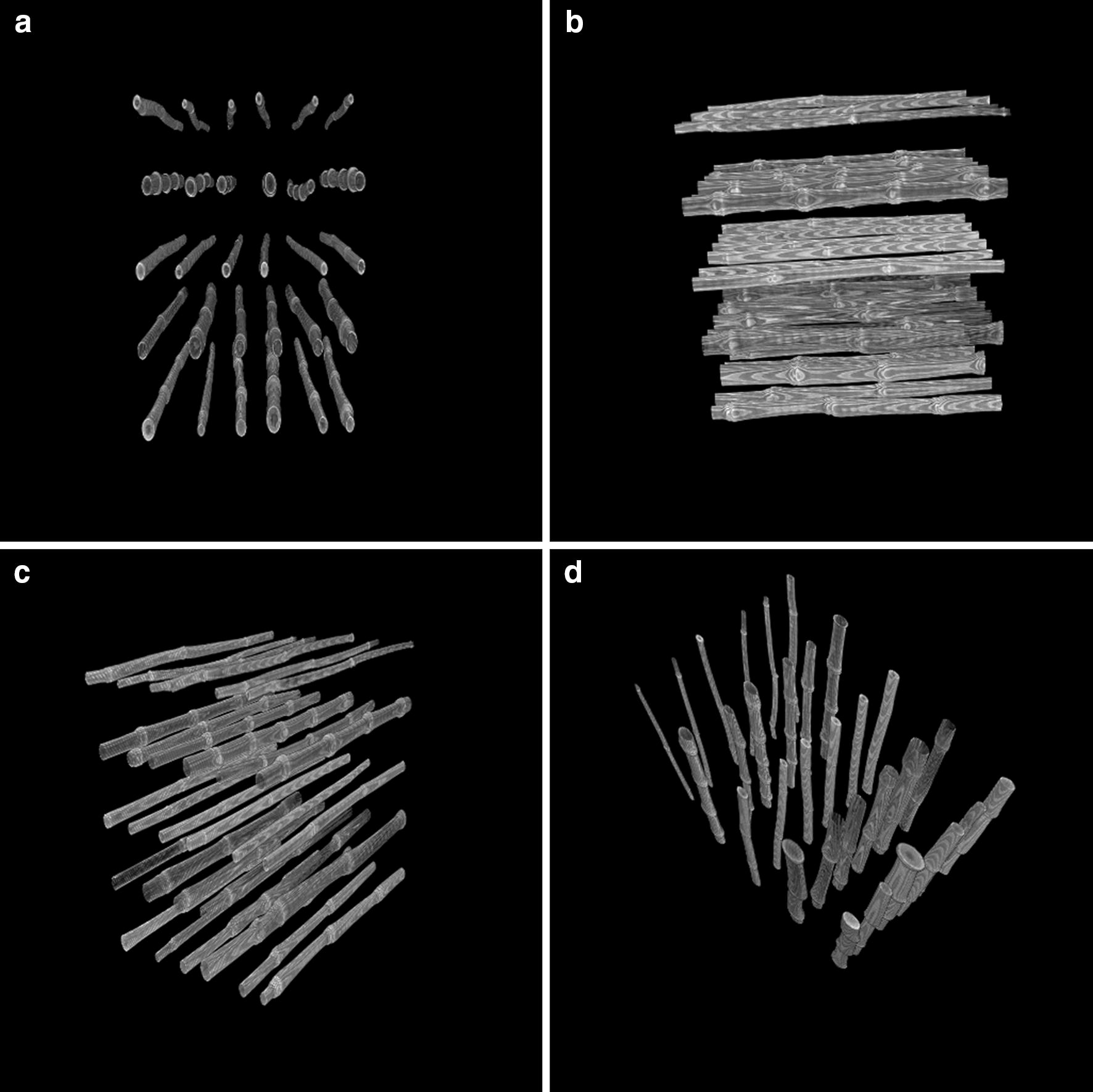
**a**–**d** Arrangement of sorghum stems on the platform viewed three-dimensionally from different angles

All X-ray imaging was performed at the Diagnostic Imaging & Cancer Treatment Center of the Texas A&M Veterinary Medicine & Biomedical Sciences facilities in College Station, Texas. A SOMATOM Definition AS+ (SIEMENS) medical CT was used using 120 kVp, 1.024 pixels per mm, at a 0.6 mm slice thickness. This medical CT scanner has a wide circular sliding gantry of ~ 75 cm wide that allows more samples per scan compared to a typical micro-CT system and is primarily used to test human or animal patients. To obtain maximum high throughput all harvested plant samples were loaded on a 5 × 6 platform in groups of 30 for trait extraction (Fig. 2).

### Morphological measurements

In Set 1, stem morphological traits were collected on the 1.5-m section scanned in the CT analysis (see Additional file 1: Table S1). Each internode was numbered, with the lower number closer to the base of the plant. The distance between each node was recorded as internode length (cm), and internode diameter (mm) was measured at the center of each internode using a digital caliper. Internode volume was estimated using the formula.

1 $$v = \pi r^{2} h$$

where *r* is the radius of the stem, and *h* is the length of the internode. Internode fresh mass was taken for each internode using a digital scale (model 95364 CEN-TECH ^®^). Internode mass density was calculated using the formula

2 $$\uprho = \frac{m}{v}$$

where, *m* is mass (g), and *v* is the volume of the internode. Samples were dried in an air-force oven at 60 °C for 1 week post phenotyping to estimate dry internode weight and density.

### Stem biomechanics

Biomechanical properties were collected on the previously described samples. All internodes were cut at the nodes and were subjected to a three-point bending test (3PBT) following the methods described in Gomez et al. [28]. Biomechanical properties were determined based on the Euler–Bernoulli beam theory since the tested internodes were relatively slender. The following formula calculated the dimensionless slenderness ratio

3 $$\uplambda = \frac{L}{D}$$

where *L* is the length of the stem section and *D* is the diameter of the stem section. A slenderness ratio > 10 was maintained on all specimens.

The second moment of an area (*I* with units mm^4^) quantifies the resistance to bending provided by cross-sectional geometry and size. The stem cross-section was approximated as a circular cross-section. For beams with a solid circular cross-sectional geometry, *I* is given by the formula

4 $$I = \frac{{\pi D^{4} }}{64}$$

where *D* is the diameter of the stem section. The geometric property for a given cross-section or section modulus (*Z*) was also calculated by

5 $$Z = \frac{I}{r}$$

where *I* is the second moment of an area and *r* is the internode radius. The elastic (Young’s) modulus *E* reported in MPa is the quotient of normal stress to normal strain throughout the linear range of elastic behavior [3], henceforth referred to as “material stiffness”. From a three-point bending test, *E* is given by

6 $$E = \frac{{F_{i} L_{in}^{3} }}{{48 B_{i} I}}$$

where *B*_*i*_ is the lateral displacement it took to bend the stem section without damaging its structural integrity. *F*_*i*_ is the force required to bend the stalk to displacement *B*_*i*_, *L*_*in*_ is the length of the stem section between the two supports, and *I* is given by Eq. (4).

Stalk strength was taken as the maximum stress required to break the structural integrity of the stem [3] and is given by

7 $$\sigma_{\hbox{max} } = \frac{{\left( {F_{u} } \right)L_{in} }}{4I}*r$$

where *F*_u_ is, the force required to induce breakage, *L*_in_ is internode length, *r* is internode radius, and *I* is the second moment of an area (Eq. 4). Flexural rigidity (herein referred to as rigidity), symbolized as *EI* (Eq. 8) represents the resistance of a beam to bending forces regarding both size, geometry and material properties (stiffness). Plants are composite materials; therefore, the calculated biomechanical properties are interpreted as spatially averaged Young’s modulus [31, 33] and effective flexural rigidity across the entire heterogeneous plant tissues [33, 34]

### Anatomical measurements

Visual stem pithiness measurements from Carvalho and Rooney [8] were used as these data included the same genotypes that were in Set 1. In brief, the percent of pithy stem cross-section area was visually estimated by using a rating scale system. This scale ranges from 1 to 9, where 1 corresponds to 90–100% pithiness and 9 to 0–10% pithiness. One unit increase in the scale equals to 10% decrease in the percent of the pithy area. For Set 2, the same protocol was followed, but in this case ratings were taken in the same plants that were scanned in the CT, and for internodes 3 and 6 only.

### Computational image analysis

**Fig. 3:**
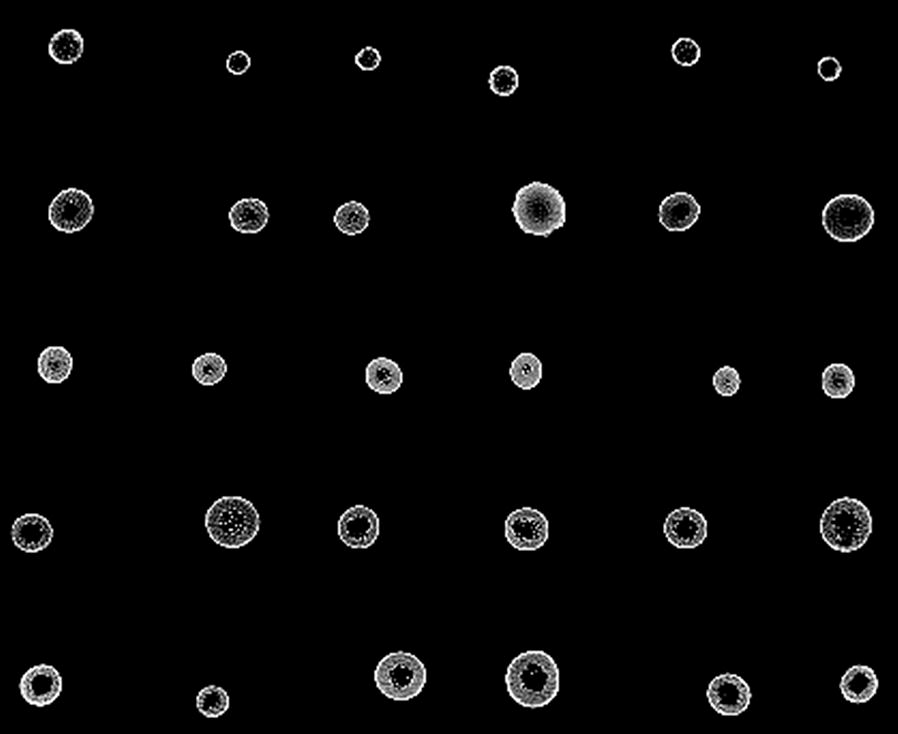
Cross sections of 30 sorghum stems during one run of CT scanning showing different attenuation levels

A customized computer program was developed by the authors in the MATLAB environment (Mathworks, Inc., Natick, MA, USA), to extract morpho-anatomical attributes from CT cross-section images (Fig. 3). For the algorithm developed, the input is an image, and output is the region centers, diameters, rind area, cross-section intensity, and percent pithy area. The process consisted of the following steps. First, we performed a morphological closing operation to connect some disconnected regions due to the image noise using a disk-shaped structuring element with radius 3 [obtained by strel (‘disk’, 3)] in MATLAB. Then, isolated regions were extracted using the MATLAB routine “regionprops”. Small regions, not part of the platform [areas there were below a preset threshold (which is image; width*image and height * 0.00005)] were excluded. Additionally, a circle was fit for each remaining region to obtain the exterior outline of each cross section. If the eccentricity for a region was too low (< 0.08) (which means the region was not a circle), this region was not included in the analysis.

Next, the cross sections outlined previously were then used to determine the center, radius, diameter, rind area, intensity, and pithy area. CT intensity was measured as the ratio of the mean pixel intensities of a region and the maximum possible intensity of the image (which is 255). The rind area was defined as the area of the outer region. The inner circle was obtained by first excluding the pixels which intensities are higher than a threshold (175), and then fit the circle using the remaining pixel locations in that region. Finally, the percent pithy area was defined as the ratio between dark pixels (intensity is below a threshold, which is 20), inside each region and the area of the entire inner circle.

In total, over six morph-anatomical attributes were determined for each cross-section. Since it was not possible to detect nodes using the algorithm, node sections of the stem were added manually to the output. A separate function estimated internode length by multiplying the slice thickness of the CT image and the number of images within an internode section.

### Image preprocessing

A total of ~ 500,000 images were produced by the CT scanning of ~ 150 plants. The preprocessing of phenotypic data involves removal of node sections since no data was collected at the nodes. Missing data was removed that may have occurred when the stems cross section move out of the area of estimation, or the algorithm did not detect a cross-section. Outlier detection was also performed by plotting the samples and identifying any extreme outliers.

### Statistical analysis

At first, individual data points estimated by the algorithm were averaged by internode. Next, the observed values for the morpho-anatomical traits were analyzed using a linear model, written as

8 $$y_{ij} = \mu + g_{i } + i\left( g \right)_{j\left( i \right)} + \varepsilon_{ij}$$

where *µ* is the grand mean, *g*_*i*_ is the fixed effect of the genotype, $i\left( g \right)_{j\left( i \right)}$ is the fixed effect of the internode number within the genotype, and *ε*_*ijk*_ is the random error component. LS Means and standard errors were estimated for each genotype using the linear model. Pearson correlation coefficients where estimated using the LS Means from the model for all manual and CT traits collected. The same model was run as a mixed model, except all terms were random using the restricted maximum likelihood method (REML) to estimate the repeatability on a plot mean basis as follows:

9 $$H^{2} = \frac{{\sigma_{G}^{2} }}{{\sigma_{G}^{2} + \sigma_{E}^{2} }}$$

where $\sigma_{G}^{2}$ is the genotypic variance, $\sigma_{E}^{2}$ is the error variance, respectively [35]. For percent pithy area, plot mean values from Set 1 were combined with plant-based values from Set 2 to estimate repeatabilities and for modelling analysis.

A univariate regression was performed for internode diameter, length, and pithiness to study the relationship between CT-derived and ground-truth stem traits. Three predictive models were fit to validate the accuracy and usefulness of the results. In the first model, the CT-derived internode diameter predicts the diameter collected manually; Diameter = Diameter-CT. In the second model, CT-derived internode length predicts the internode length collected manually; Length = Length-CT. In the third model, the CT-derived internode percent pithy area predicts the visual pithiness ratio; Pith = Pith-CT. The performance of the models from the univariate regression was assessed by the leave-one-out cross-validation (LOOCV) method. For this, the data was split into two parts, and a single observation (*x*_1_*, y*_1_) was used for the validation set, and the remaining observations {(*x*_2_*,y*_2_),…,(*x*_*n*_*,y*_*n*_)} made up the training set. The statistical learning method was fit on the n-1 training observations and a prediction $\hat{y}$ was made on the excluded observation using its value *x*_1_. The root mean square error (RMSE) of prediction was estimated for each validation, RMSE_1_, RMSE_2_, …, RMSE_k_:

10 $$RMSE = \sqrt {\frac{1}{n} \sum \left( {y - \hat{y}} \right)^{2} }$$

where y and $\hat{y}$ are the observed and predicted values in the model. The model acceptability was assessed on the average of these n test error estimates.

The models were further evaluated by plotting predicted versus observed values in a 1:1 diagram of the model identified from the LOOCV method with the lowest RMSE. An R^2^ value close to 1.0 with a slope of observed versus predicted close to 1.0 and small RMSE values indicate that the model is precise with little bias [36]. All statistical analyses were implemented in the R statistical language and computing environment [37].

## Results

### Phenotypic variation for CT estimated traits was detected

**Fig. 4:**
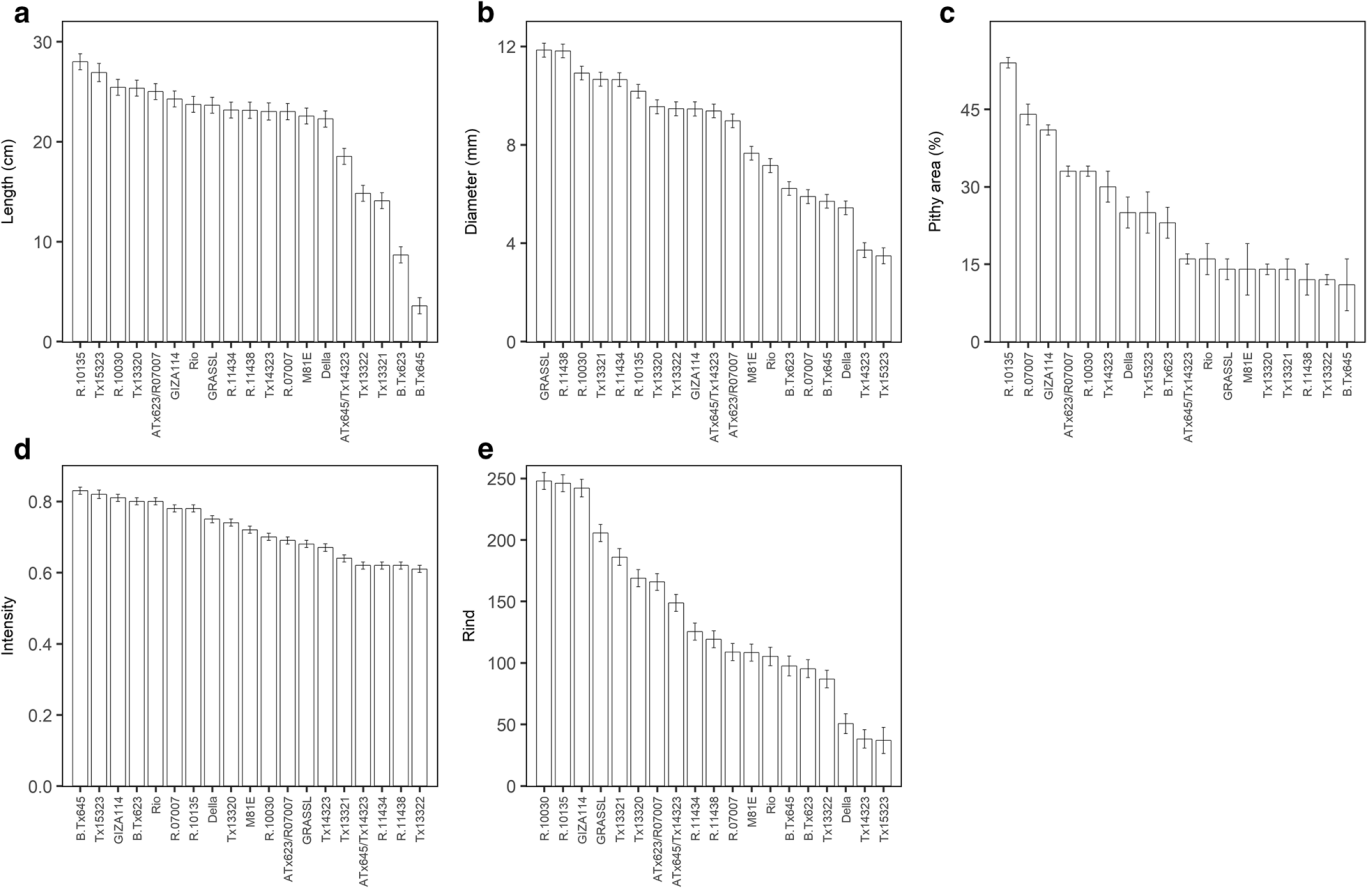
Phenotypic variation for morpho-anatomical traits for 19 genotypes from Set1. Genotypes have been sorted by specific trait. The vertical bars indicate the relevant standard error; **a** length (cm), **b** diameter (mm), **c** pithy area (%), **d** intensity, **e** rind area

Phenotypic variation existed among genotypes for all CT-derived traits. In Set 1, the genotypic LS-means across all internodes and plants ranged from 3.6 to 28 pixels for internode length; stem diameter ranged from 3.5 to 12 pixels; stem pixel intensity ranged from 0.61 to 0.83, and stem rind area ranged from 37 to 248 pixels. In the entire panel (Set 1 and Set 2 together), percent pithy area ranged from 11 to 60% on a mean genotype basis. The CT estimates effectively identified groups of genotypes with common phenotypes (Fig. 4). On average, the late maturing genotypes had the largest internode length, diameter, and rind values (see Additional file 1: Table S1, Fig. 4a, 4b, 4e). Earlier genotypes showed higher stem intensity values (see Additional file 1: Table S1, Fig. 4d) with a few exceptions, notably Tx14323, and GIZA114.

### Repeatability for CT estimates trait

Repeatability estimates for CT-derived traits ranged from 0.51 to 0.72 and repeatabilities for manually collected traits ranged from 0.66 to 0.85 (Table 1). In most cases, the CT-estimated traits were lower than the ground-truth trait estimates. High repeatabilities (~ .70) were observed for rind, diameter, and volume, followed by length and intensity at 0.61, and by percent pithy area and second moment of an area, at 0.56 and 0.51, respectively. Overall, *H*^2^ for CT and manually collected data were consistent, with one notable exception. For percent pithy area, repeatability values for CT estimation were 1.5 times lower than visual measurements.

**Table 1:** Repeatabilities for CT-derived traits and ground-truth traits measured in 29 diverse sorghum genotypes

Trait	CT	Manually collected
	H^2^	H^2^
Internode length	0.61	0.76
Internode diameter	0.70	0.81
Internode volume	0.71	0.83
Second moment of an area (*I*)	0.51	0.66
Intensity	0.61	NA
Pithiness	0.56^a^	0.85^a^
Rind	0.72	NA

*NA*, data was not collected manually for this trait

^a^Measured at individual plant basis

### Accuracy of estimating morph-anatomical traits using X-ray CT in sorghum

**Fig. 5:**
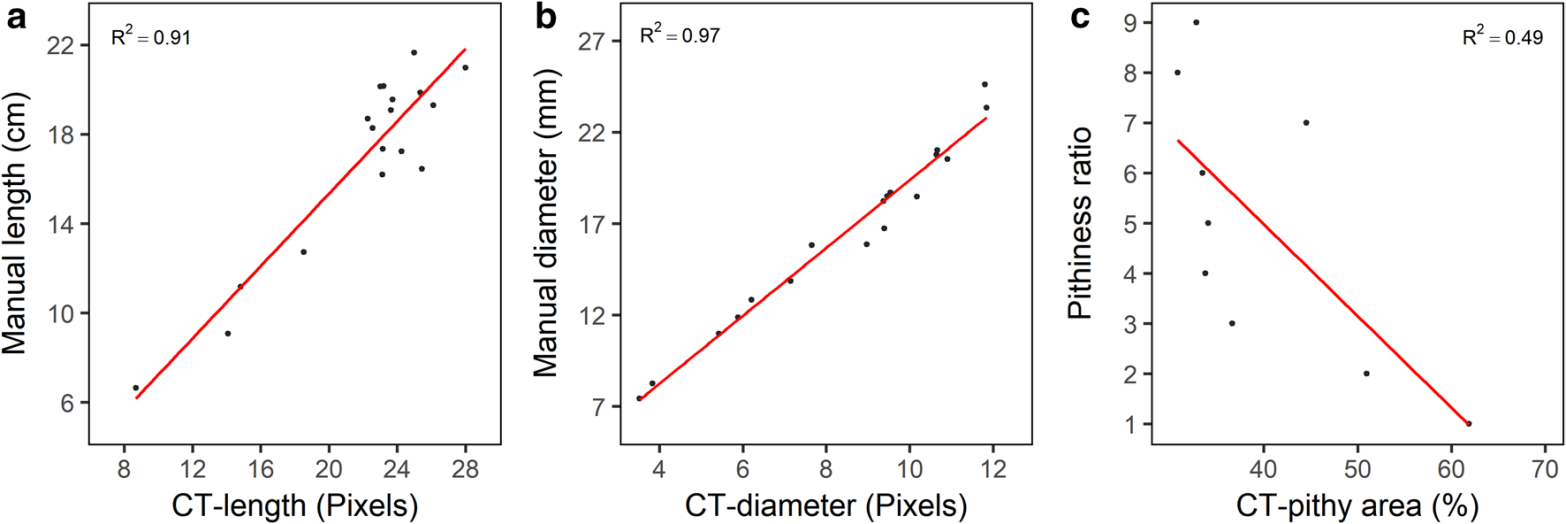
Association between CT and manually collected traits for 29 sorghum genotypes; **a** length, **b** diameter, **c** pithy area (%)

The coefficients of determination of genotypic CT mean values regressed to genotypic ground-truth means were high for morphological traits and moderate for one anatomical trait (Fig. 5). For length and diameter, the R^2^ were 0.91 and 0.97, respectively. For percent pithy area, the R^2^ was 0.49. The leave-one-out cross-validation analysis (LOOCV) applied at the individual internode data point basis (genotype vs. plant vs. internode) revealed average R^2^ and RMSE values of 0.56 and 3.75 for internode length, respectively. For internode diameter, an average R^2^ of 0.54 and an average RMSE of 2.78 was observed. For pithiness ratio, the average R^2^ was equal to 0.44 while an average RMSE of 1.63 was found.

**Fig. 6:**
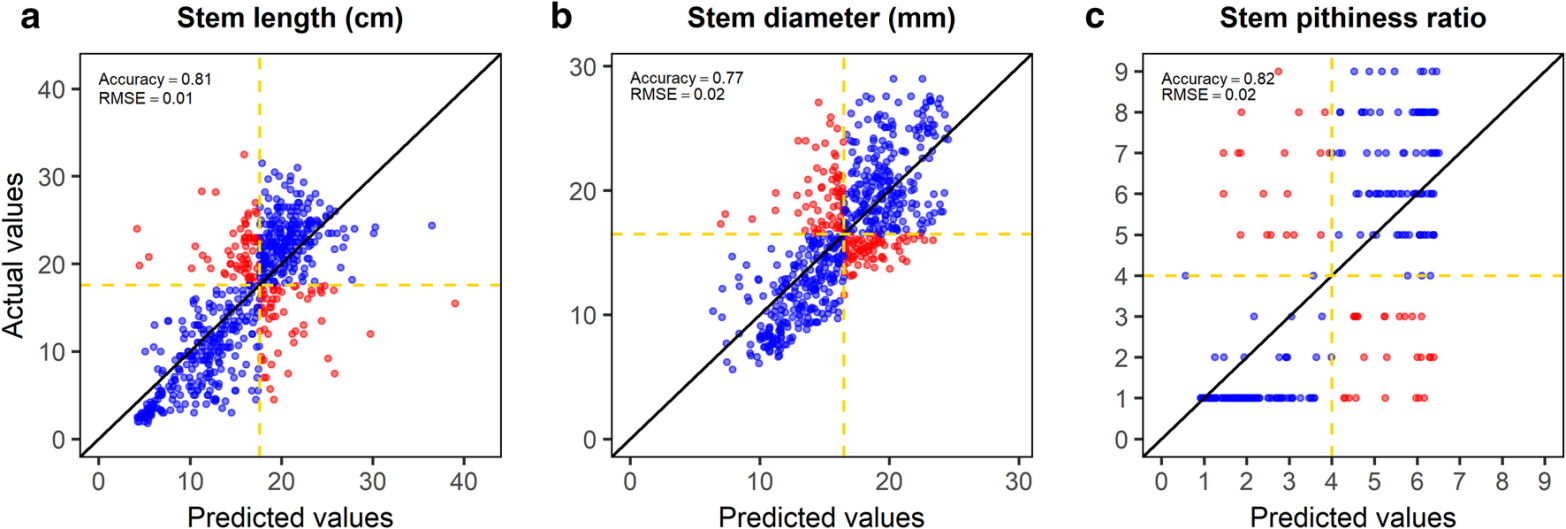
Predicted versus observed plots for three traits and the model with the lowest RMSE select using the LOOCV method. Yellow line depicts a 50% cut off; **a** stem length, **b** stem diameter, **c** stem pithiness ratio

The adequacy of both models was assessed by plotting predicted versus observed (manually phenotyped) internode length, stem diameter, and pithiness ratio for all observations. Figure 6 shows the observed and predicted values for the model with the lowest RMSE selected from the LOOCV. The lowest RMSE for stem length, stem diameter and stem pithy area were 0.01, 0.02, and 0.02, respectively. The values for all three models were relatively precise and accurate across all observations. Furthermore, a 50% cut off line was added to evaluate the model as a selection tool in a sorghum breeding program (Fig. 6). The cut off separated the plots into four quadrants. The quadrants with the blue observations were classified as the individual internodes that would be correctly classified using the model selected for each of the three traits. The accuracy of the model on classifying the values for the internodes of each genotype for stem length, diameter, and pithiness ratio was 81, 77, and 82%, respectively (i.e., from the total number of data points predicted, 81, 77, and 82% would be classified correctly upon selection).

### Correlations among CT-derived traits

**Fig. 7:**
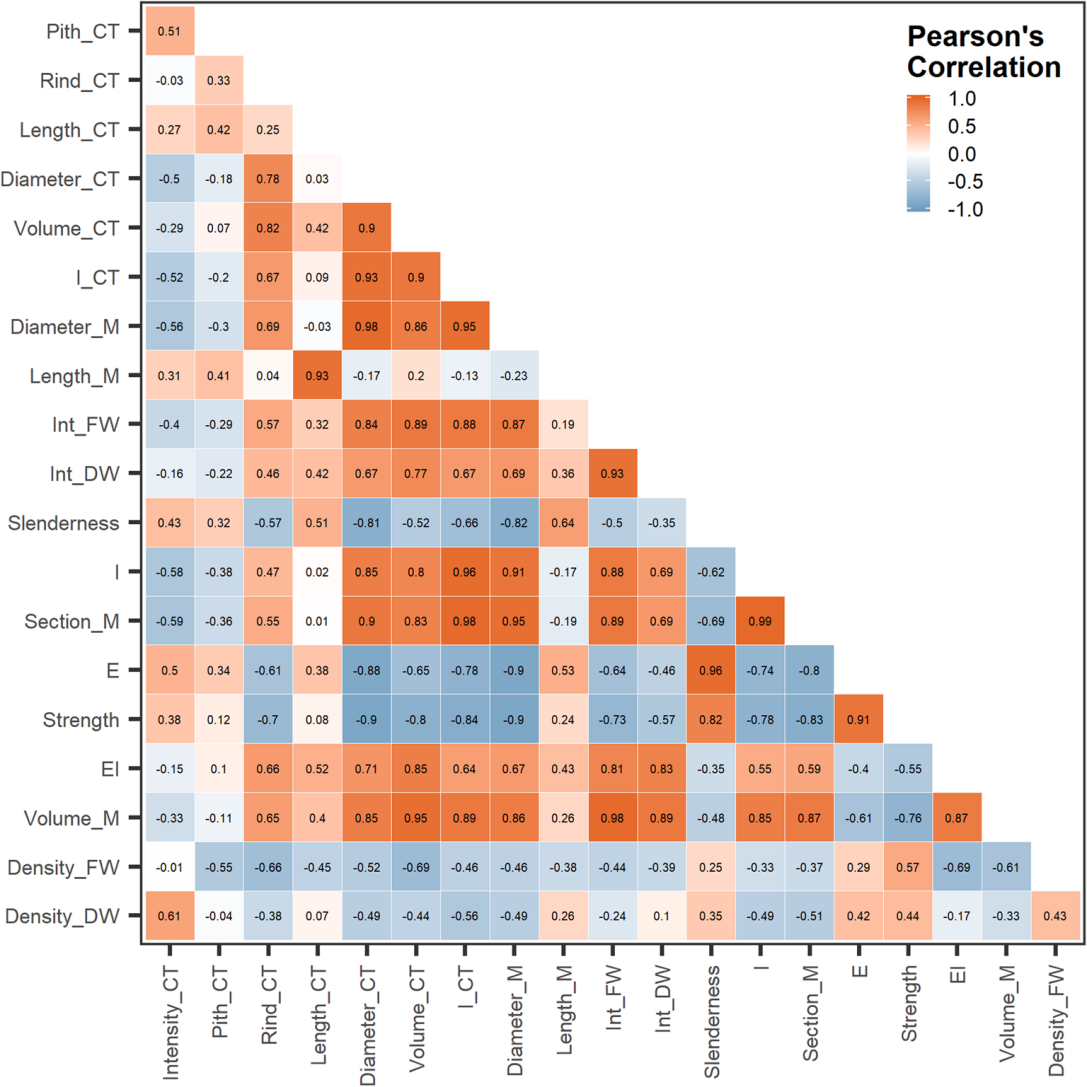
A heatmap depicting Pearson’s correlation coefficient for all traits collected in 19 sorghum genotypes from Set1

Correlations between CT-derived traits and morpho-anatomical manually collected traits were variable (Fig. 7). For example, CT and ground truth measurement of internode volume, internode fresh weight, and dry weight were highly correlated. As expected, stem intensity was correlated with internode dry weight density (*r *= 0.61; P < 0.01), but pith-CT had very low correlations with the manually collected measurements. Rind-CT had a moderate correlation with section modulus, rigidity, and a high correlation with volume-CT; (*r *= 0.55; P < 0.001), (*r *= 0.66; P < 0.005), (*r *= 0.82; P < 0.0001).

Results demonstrate that CT estimates of morphological traits best correlated with biomechanical properties. Volume-CT was highly correlated with rigidity (*r *= 0.85; P < 0.001), respectively. Diameter-CT was positively correlated with rigidity (*r *= 0.71; P < 0.001) and negatively correlated with strength and stiffness (*r *= − 0.9; P < 0.001), (*r *= − 0.88; P < 0.001). These findings are consistent with results found by Gomez et al. [28].

## Discussion

**Fig. 8:**
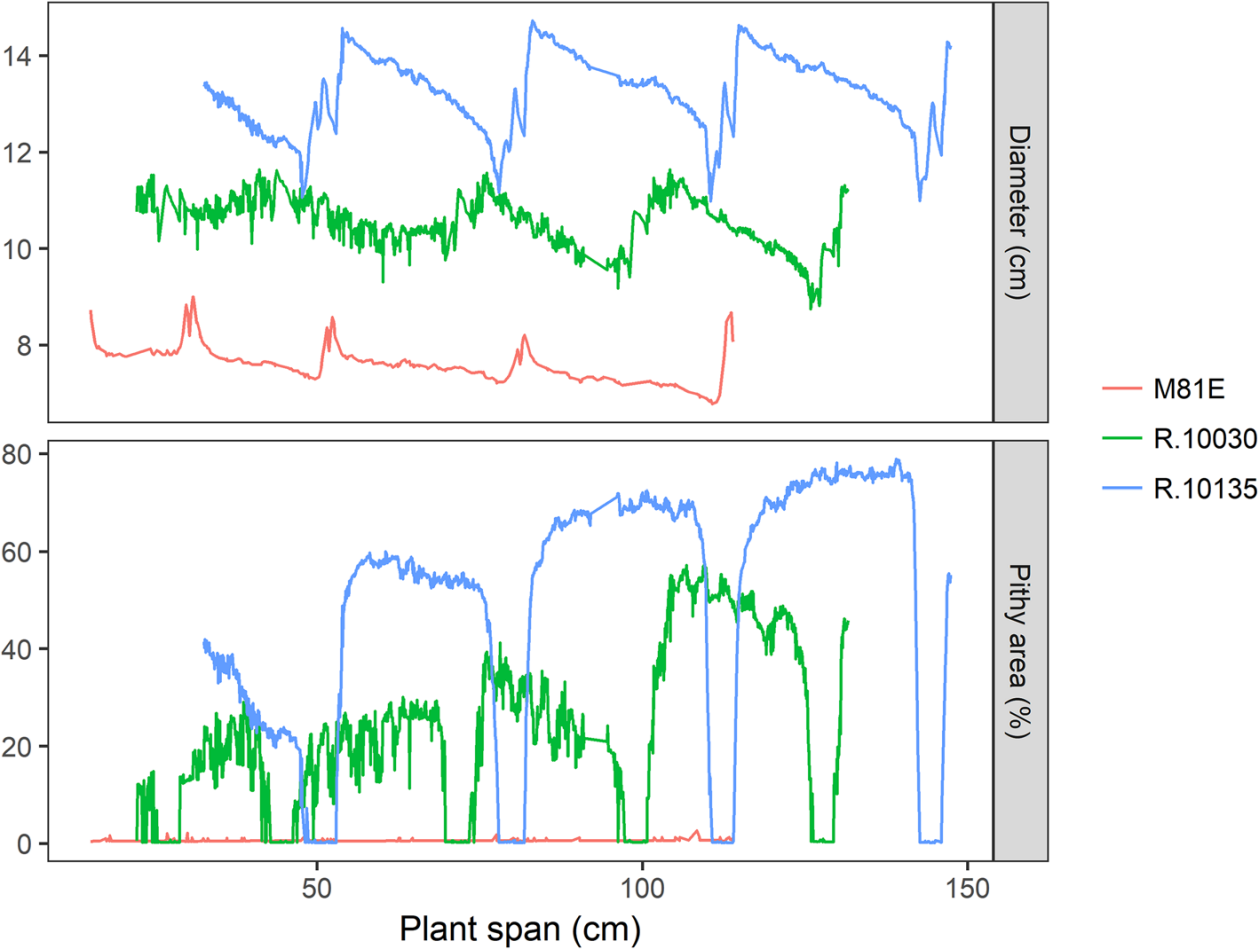
Raw data from diameter and pithy area collected by CT for three sorghum genotypes plotted across the span of the plant stem

The high-throughput digital phenotyping imaging pipeline presented herein was able to reduce human input, time and accurately detect variation for important morpho-anatomical traits in well-characterized sorghum genotypes at sounding repeatability rates. The magnitude of the repeatability is a major factor in determining the efficiency and relevance of any phenotyping methodology in a germplasm screening program. In our case, repeatability estimates for CT-derived traits were moderate and were lower than ground-truth measurements. The differences are likely due to minute variation present along the plant stem that could be captured by the CT method (Fig. 8) which cannot be assessed manually or visually. As such, a single point measurement is likely to misrepresent the natural variation of each internode, which might cause an overestimation of variance when assessed across different plants and genotypes. Errors associated with the algorithm estimation might also be a complicator, but for this case, improvements are possible.

Our image analysis pipeline could identify genotypes with superior morpho-anatomical traits that were consistent with ground-truth based classification previously performed by Carvalho and Rooney [8] and Gomez et al. [28]. For example, the genotype Rio had smaller stem diameter than the genotype Tx13321 but longer internodes than Tx13321. Interestingly, the CT-estimated traits (internode diameter, internode length, and percent pithy area) were moderate to highly predictive of the manually collected traits. Percent pithy area explained 50% of the variation for pithiness rating. This value was much lower than the coefficient of determination found for the other traits evaluated. Stem pithiness occurs when the stem parenchyma cells die and are gradually filled with air creating a white, cottony, and pithy tissue. CT-derived percent pith is measured at every 0.6 mm slices across the length of the stem that can contribute to a larger variation than one visually collected pith rating. Therefore, one visual rating may not explain the variation across the length of the internode collected by the CT-based measurement and may have contributed to the lower R^2^ observed for stem pithiness in this study. Although the low variance explained for our pithiness rating may be a result of the visual score, visual ratings are one method to rate pithiness, and it has been demonstrated that using a flatbed scanner can increase the accuracy of phenotyping [8]. Therefore, an association between CT-derived percent pith and percent pith area estimations using a flatbed scanner might result in a higher association.

In this study, CT estimated morphological traits had the strongest correlations with mechanical properties. This finding is consistent with a computation sensitivity analysis in maize by von Forell et al. [27], demonstrating that morphological traits demonstrate a stronger association with mechanical traits rather than tissue or material properties. The results from the computation sensitivity analysis were also consistent with a study using a micro-CT in maize [15]. In our study, morphological measurements also had a strong effect on mechanical properties. For example, CT estimates of the second moment of an area were negatively correlated with stem strength and stiffness, demonstrating the strong effect stem morphology may have on mechanical traits. Similar results were reported by Gomez et al. [28]. Furthermore, rind-CT was moderately associated with section modulus and is in line with a study by Ookawa et al. [38] where an *indica* variety of rice had strong culms due to a large section modulus that is associated with stem wall thickness. These results indicate that stem morphology has a strong effect on mechanical properties and morphological traits such as the second moment of an area and section modulus are to be considered when selecting for lodging resistance.

CT is based on the principle that the density of the tissue passed through by the X-ray beam can be measured by calculation of the attenuation coefficient [39]. Therefore, material density is a major factor to consider when running plant samples in a CT scanner, as plant organs vary in tissue density. X-ray attenuation is mainly determined by the material properties of the plant tissues and can become visible by contrast according to density and atomic number of elements [12, 40]. Differences in X-ray attenuation in several plant stems were visibly apparent and primarily dependent on the anatomy, composition, and material density of the cross-section of the stem (i.e. rind is more lignified) (Fig. 3). At this attenuation level obtained by the SOMATOM Definition AS+ medical CT it is possible to detect the material density of the stems as well as rind and pithy area. It has been shown that medical CT scanners capture the changes in material density and composition of relative light and large objects [39, 41], such as stems of grasses. In this study, stem ‘density’ was estimated as the pixel intensities of a region and had a high correlation with internode dry weight density. Other studies indicate similar results [41]. Intensity as used in this study, is a new method to quantify stem density in sorghum or other grass stems. Furthermore, in a recent study by the authors, it was found that internode density, volume, and stiffness can predict strength and can explain 75% of the variation [42]. Therefore, using the methods developed in this study in combination with biomechanics can be used to apply selective breeding tools to improve lodging resistance.

The need for a high-throughput method for quantifying important morpho-anatomical traits related to stem lodging and juice yield in bioenergy sorghum motivated this pipeline and platform. While many high throughput methods are being developed to phenotype plants using unmanned aerial vehicle (UAV), robotics, and high throughput platforms such as the ARPA-E TERRA-REF project (http://terraref.org/) [43–46], none of these methods allow for combining external and internal stem phenotypic information of plants. Besides the potential applications discussed for our pipeline, it can also be applied to produce highly dimensional data used in 3D reconstruction and crop modeling (Additional file 2: Video S1).

Computer vision is an active and challenging field of computer science that is rapidly providing tools applicable to biological problems. In principle, images can be mined for phenotypes other than those which were collected [47]. The spatial scanning resolution in an X-ray CT depends on the spot size of the X-ray source, the resolution of the X-ray detector, and used magnification of the system [14]. While adding multiple samples in the medical CT may have introduced noise into the later measurements, we found that the image algorithm developed in this study was able to detect and extract useful external and internal phenotypic information effectively and accurately. However, the work herein is preliminary; there is room to improve on both processing and algorithms. For example, some of the coefficients of determination of the univariate regression did not explain all of the variation. We believe this was because plant stems vary in tissue density and the algorithm did not detect all cross-sections, therefore, it did not estimate all objects in the CT. Another reason may be that CT measurements were more precise and capture a larger portion of the variation than one single manually collected point that can also be subjective. We believe using computer vision and machine learning methods are warranted in future studies using CT in order to enable accurate phenotyping. In summary, the results indicate that medical CT scans can produce useful data in significantly reduced times, making it a good alternative for phenotyping plants.

## Conclusions

The results herein indicate that CT-based estimates are associated with important traits in bioenergy/forage sorghum. Furthermore, predicting traits such as stem length, diameter, and pithiness ratio at the internode level by utilizing a high-throughput digital phenotyping approach using CT appears possible in an applied breeding program. Further work to improve algorithms and the accuracy of our models will enhance the speed and efficiency of this methodology allowing it to be applied to large populations, panels, and hybrids with high fidelity. As a selection tool, our protocol appears readily applicable in field-based and large-scale breeding programs.

## Additional files

**Additional file 1: Table S1.** Sorghum genotypes used in this study with their respective set, maturity, type, and end-use.

**Additional file 2: Video S1.** Animation of the 3D model of a group of 30 sorghum stems during one run of CT scanning.
