# Supplementary material for: High throughput phenotyping of morpho-anatomical stem properties using X-ray computed tomography in sorghum
Source: Plant Methods. 2018 Jul 13;14:59. doi: 10.1186/s13007-018-0326-3 (PMC6043981; doi:10.1186/s13007-018-0326-3)
Supplement: Supplementary file 1 — Additional file 1: Table S1. Sorghum genotypes used in this study with their respective set, maturity, type, and end-use. [file 13007_2018_326_MOESM1_ESM.docx]

**High throughput phenotyping of morpho-anatomical stem properties using X-ray computed tomography in sorghum**

Francisco E. Gomez ^1,2*^, Geraldo Carvalho Jr ^2^, Fuhao Shi ^3^, Anastasia H. Muliana ^4^ and William L. Rooney ^2^

^1^North Carolin State University, Department of Crop Sciences and Soil Sciences, Raleigh, NC 27695-7620, USA

^2^Texas A&M University, Department of Soil and Crop Sciences, 370 Olsen Blvd, College Station, TX 77843, USA

^3^Texas A&M University, Department of Computer Science and Engineering, 3112 TAMU, 710 Ross St, College Station, TX 77843, USA

^4^Texas A & M University, Department of Mechanical Engineering, 401 Joe Routt Blvd, College Station, TX, 77843, USA

Key words: high-throughput phenotyping, computed tomography imaging, Sorghum bicolor, bioenergy

Geraldo Carvalho Jr: gacjunior@gmail.com

Fuhao Shi: fuhaoshi@gmail.com

Anastasia H. Muliana: amuliana@tamu.edu

William L. Rooney: wlr@tamu.edu

*Corresponding author

E-mail: fegomez@ncsu.edu

**Table S1**. Sorghum genotypes used in this study with their respective set, maturity, type, and end-use.

| **Genotype** | **Set** | **Maturity** | **Type** | **End-Use** |
| --- | --- | --- | --- | --- |
| B.Tx623 | 1 | PI | Inbred Line | Grain |
| B.Tx645 | 1 | PI | Inbred Line | Grain |
| Della | 1 | PI | Inbred Line | Biofuel |
| Tx14323 | 1 | PI | Inbred Line | Forage |
| Tx15323 | 1 | PI | Inbred Line | Forage |
| R.07007 | 1 | PS | Inbred Line | Biomass |
| Tx13320 | 1 | PS | Hybrid | Forage/Biofuel |
| M81E | 1 | PS | Inbred Line | Biofuel |
| Rio | 1 | PS | Inbred Line | Biofuel |
| Tx13321 | 1 | PS | Hybrid | Biofuel |
| Tx13322 | 1 | PS | Hybrid | Biofuel |
| ATx623/R07007 | 1 | PS | Hybrid | Biomass |
| ATx645/Tx14323 | 1 | PS | Hybrid | Forage/Biomass |
| R.11434 | 1 | PS | Inbred Line | Biomass |
| R.11438 | 1 | PS | Inbred Line | Biomass |
| R.10030 | 1 | PS | Inbred Line | Biomass |
| R.10135 | 1 | PS | Inbred Line | Biomass |
| GRASSL | 1 | PS | Inbred Line | Biomass |
| GIZA114 | 1 | PS | Inbred Line | Biomass |
| (GIZA114/Umbrella)-101 | 2 |  |  |  |
| (GIZA114/Umbrella)-102 | 2 |  |  |  |
| (GIZA114/Umbrella)-103 | 2 |  |  |  |
| (GIZA114/Umbrella)-104 | 2 |  |  |  |
| (GIZA114/Umbrella)-105 | 2 |  |  |  |
| (GIZA114/Umbrella)-111 | 2 |  |  |  |
| (GIZA114/Umbrella)-112 | 2 |  |  |  |
| (GIZA114/Umbrella)-113 | 2 |  |  |  |
| (GIZA114/Umbrella)-114 | 2 |  |  |  |
| (GIZA114/Umbrella)-115 | 2 |  |  |  |
